# Molecular analysis and geographic distribution of the recent Indonesian rabies virus

**DOI:** 10.14202/vetworld.2023.2479-2487

**Published:** 2023-12-25

**Authors:** Nirma Cahyanti, Sumaryati Syukur, Endang Purwati, Yul Fitria, Ibenu Rahmadani, Didik T. Subekti

**Affiliations:** 1 Biotechnology Program Postgraduate School, Andalas University, West Sumatra Province, Indonesia; 2 Faculty of Mathematics and Natural Sciences, Division of Chemistry, Department of Biotechnology, Andalas University, West Sumatra Province, Indonesia; 3 National Reference Laboratory for Animal Rabies - Animal Disease Investigation Center of Bukittinggi, Bukittinggi, Indonesia; 4 Center for Biomedical Research, Research Organization for Health, National Research and Innovation Agency, Cibinong Science Center, West Java Province, Indonesia; 5 Indonesian Research Center for Veterinary Science, Agency for Agricultural Research and Development, Indonesian Ministry of Agriculture, Bogor, West Java Province, Indonesia

**Keywords:** genetic diversity, geographical distribution, nucleoprotein gene, rabies

## Abstract

**Background and Aim:**

Some Indonesian islands, including Sumatra, Kalimantan, Sulawesi, Java, and East Nusa Tenggara, have endemic rabies. Rabies outbreaks in Bali began from 2008 to 2011 and continue to occur sporadically. This study aimed to study the molecular analysis and geographical distribution of Indonesian rabies virus (RABV) from 2016 to 2021 and compare to previous periods.

**Materials and Methods:**

Virus isolates from 2016 to 2021 were extracted from dog brains and sequenced at the nucleoprotein gene locus. They were compared with data sequences available in the GenBank database. Indonesian RABV from the previous three periods (before 1989, 1997–2003, and 2008–2010) was extracted from the GenBank database. The genetic diversity in this study was based on the N gene of Indonesian RABV.

**Results:**

Asian RABV, which is genetically close to the Indonesian virus, is a virus from China (ASIA-3 cluster) and from the Southeast Asia region, namely, virus isolates from Sarawak and Malaysia and some Cambodian isolates. Rabies virus, which was isolated from the Bali islands, was the new cluster first detected and published in Bali, Indonesia, in 2008, while RABV from West Sumatra Province, which was isolated from 2016 to 2021, was also considered a new cluster that is genetically distant from other clusters in Indonesia.

**Conclusion:**

The RABV in Indonesia is divided into five clusters. The isolates from West Sumatra Province from 2016 to 2021 were a new cluster genetically distant from other Indonesian viruses.

## Introduction

Rabies is the oldest zoonotic disease in the world and is caused by the rabies virus (RABV) of the *Lyssavirus* spp. [1, 2]. Most human deaths from rabies have occurred in Asia and Africa at 56% and 44%, respectively [3–5]. Global human deaths due to rabid dogs are estimated at more than 55,000 [4], with an annual reported incidence of 30,000 in Asia and 23,000 in Africa [1].

In Asia, several countries have endemic rabies, such as India, Bangladesh, Philippines, Thailand, Indonesia, Myanmar, China (mainland China), and Korea [5–7]. Several districts and countries, including Hong Kong, Taiwan, Japan, and Malaysia, have been declared free of rabies, but sporadically, there are still cases of rabies in both animals and humans [6, 8]. Not all areas in Indonesia are endemic to rabies, but only some parts of the islands of Sumatra, Kalimantan, Sulawesi, and Flores [9]. However, outbreaks of rabies in animals and humans have been reported on two islands previously known as rabies-free zones, namely, Bali and Sumbawa Island. The rabies outbreak in Bali occurred in 2008–2011 [10–12], and cases were still found until 2016 [13]. A rabies outbreak on the Sumbawa Islands occurred in 2018–2019 [14]. Recently, a new isolate and characterization of the RABV has been isolated in West Sumatra. However, its genetic diversity has not been analyzed or compared with previously reported Indonesian isolates.

In general, the RABV genome encodes five structural proteins, namely, nucleoprotein (N), phosphoprotein (P), matrix protein (M), glycoprotein (G), and RNA-dependent RNA polymerase (L), which are sequentially arranged as 3’-N-P-M-G-L-5’ [8, 15–17]. Among these five genes, there are different degrees of sequence conservation in the order of nucleoprotein > matrix protein > glycoprotein > phosphoprotein [15]. The N gene is the most conserved, so it is possible to accurately distinguish virus strains by analyzing their genetic differences [16]. Therefore, the N gene is very suitable as a basis for a molecular epidemiological study of rabies and its origin, genetic diversity, genetic identification of viral lineages, sublineages, and variants and as an analysis of their geographic distribution [8, 17].

No previous reports have analyzed or reviewed the genetic diversity of all existing Indonesian RABV isolates. The published Indonesian RABV isolates were grouped into three different isolation periods. The first period was the Indonesian RABV, which was isolated in 1989 [18]. The second period was 1997–2003 [9], and the third was Indonesian RABV in 2008–2010 [10–12]. The last period (fourth period) included the virus recently isolated during 2016–2021 in this study.

This is the first comprehensive study to analyze the genetic diversity and geographic distribution of all Indonesian RABV isolates from the four study periods. The genetic diversity in this study was based on the N gene of Indonesian RABV.

## Materials and Methods

### Ethical approval

No live dogs or cats were used in this study. This study does not require ethics approval as per the institutional ethical guidelines. All samples examined were collected from brain tissue sent by the Regional Livestock and Animal Health Offices from several districts of West Sumatra Province to the Pathology and Virology Laboratory at the Disease Investigation Center of Bukittinggi. They were subjected to laboratory analyses to detect RABV.

### Study period and location

This study was conducted from October 2021 to August 2022 in the Virology Laboratory, Animal Disease Investigation Center of Bukittinggi.

### Study design

This study involved all published Indonesian RABV isolates that have been recorded in the GenBank and the recent isolates that have been isolated. The recent isolates of nine Indonesian RABV originating from brain tissue samples were sent by the Regional Livestock and Animal Health Offices from several districts in West Sumatra Province to the Pathology and Virology Laboratory at the Bukittinggi Veterinary Center from 2016 to 2021. Each sample included eight RABV isolates from dogs and one RABV isolate from cats. The isolates were taken from nine districts in West Sumatera Province (Solok Selatan-932/INA/Dog/21, i.e., isolated from a dog in 2021 from Solok Selatan District, Agam-588/INA/Dog/21, i.e., isolated from a dog in 2021 from Agam District, Limapuluh Kota-660/INA/Dog/21, i.e., isolated from a dog in 2021 from Lima Puluh Kota District, Pariaman-550/INA/Dog/21, i.e., isolated from a dog in 2021 from Pariaman District, Bukittinggi-477/INA/Dog/21, i.e., isolated from a dog in 2021 from Bukittinggi District, Kota Solok-429/INA/Cat/19, i.e., isolated from a cat in 2019 from Kota Solok District, Pasaman Barat-267/INA/Dog/21, i.e., isolated from a dog in 2021 from Pasaman Barat District, Padang Panjang-816/INA/Dog/16, i.e., isolated from a dog in 2016 from Padang Panjang District, and Padang Pariaman-552/INA/Dog/20, i.e., isolated from a dog in 2020 from Padang Pariaman District). All brain tissues previously tested positive based on the fluorescent antibody technique, direct rapid immunohistochemical test, and mouse inoculation test conducted at the Bukittinggi Veterinary Center as a national reference laboratory for rabies.

### RNA extraction and reverse transcription–polymerase chain reaction (RT-PCR)

According to the manufacturer’s instructions, total RNA was extracted from brain tissue homogenates using Direct-Zol™ RNA Miniprep Plus reagent (Zymo Research, USA). The extracted RNA was then used for complementary DNA synthesis using the SuperScript III First-Strand Synthesis System kit (Invitrogen, USA). N gene amplification was performed through RT-PCR with forward primers (5’ACGCTTAACAACAAAAY CADAGAAG-3’) and reverse primers (5’GGRTTG ACGAARATCTTGCTCAT-3’). Amplification was performed using Phusion™ High-Fidelity DNA Polymerase (Thermo Scientific, USA). The PCR protocol was performed with initial denaturation at 98°C for 30 s, followed by 35 cycles of denaturation at 98°C for 10 s, then annealing at 52°C for 30 s, and polymerization at 72°C for 45 s. The amplicon size of the N gene was 1.536 bp.

### Sequencing and genetic analysis

Amplicons were electrophoresed using a Scie-Plas Horizontal electrophoresis system (Scie-Plas, UK) and visualized using a Firereader-V10 Plus 26M gel documentation system (Uvitec, UK) to determine the nucleotide length of the amplicon. The amplicons were then sent to 1^st^ Base-Axil Scientific Pte. Ltd. (Singapore) for sequencing.

This study involved 593 sequences of the N gene consisting of 507 sequences of RABV originating from four continents and 86 sequences of RABV originating from Indonesia. All N gene sequences of RABV were downloaded from the GenBank database, except for the nine most recent Indonesian RABV isolates. Phylogenetic analysis was performed using the neighbor-joining method using MEGA version 11 (https://www.megasoftware.net/) [19] and visualized using iTOL version 6 (https://itol.embl.de/) [20]. The potential of high-risk contact networks to predict the most probable transmission pathways was analyzed using MicrobeTrace version 0.8.3 (https://microbetrace.cdc.gov/MicrobeTrace/) [21].

The N gene sequences used in this study came from various parts of the world, such as the Americas, namely, North America (USA, Canada, Mexico, and Costa Rica) and South America (Argentina, Brazil, Colombia, and Peru). The European continent consists of Eastern Europe (Hungary, Poland, and Russia), Western Europe (Austria, France, and Germany), and Southern Europe (Slovenia). The African continent includes Middle Africa (Congo), Northern Africa (Morocco), Southern Africa (South Africa), and Western Africa (Mauritania). The Asian continent includes Western Asia (Israel), Southern Asia (Afghanistan, Iran, India, Nepal, and Sri Lanka), Eastern Asia (China, Taiwan, Mongolia, South Korea, and Japan), and Southeast Asia (Indonesia, Malaysia, Cambodia, Laos, Myanmar, Thailand and, Philippines).

## Results

### Genetic diversity and geographic distribution of Indonesian RABV

**Figure-1:**
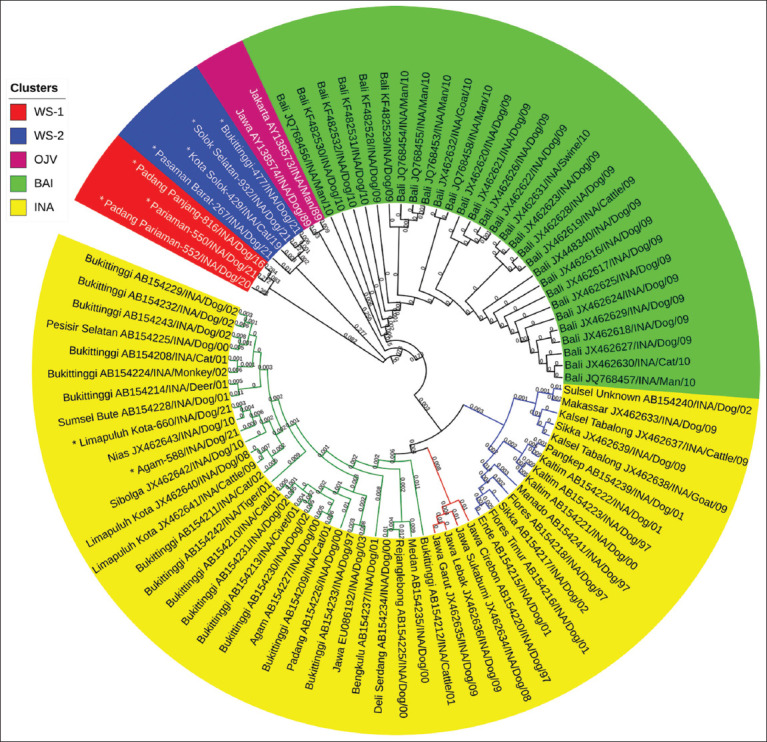
Cladogram of 86 Indonesian rabies virus strains based on the nucleoprotein (N) gene sequences. Asterisk (*), nine isolates were used in this study.

**Figure-2:**
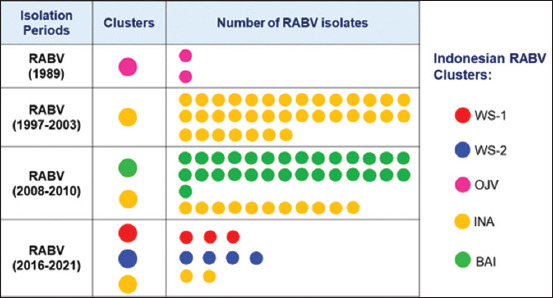
Clusters of Indonesian rabies virus from four different isolation periods based on the N gene sequences.

Indonesian RABV is generally grouped into five clusters, namely, WS-1, WS-2, OJV, BAI, and INA (Figures-1 and 2). WS-1 and WS-2 are RABV isolates from West Sumatra Province, which were isolated from 2016 to 2021. In total, 77.8% (7/9) of RABV isolated in 2016–2021 showed genetic relatedness that differed from the existing RABV clusters in Indonesia; therefore, they were considered new clusters. The remaining 22.2% (2/9) were in the INA cluster, with other viruses isolated in 1997–2003 and 2008–2010.

**Figure-3:**
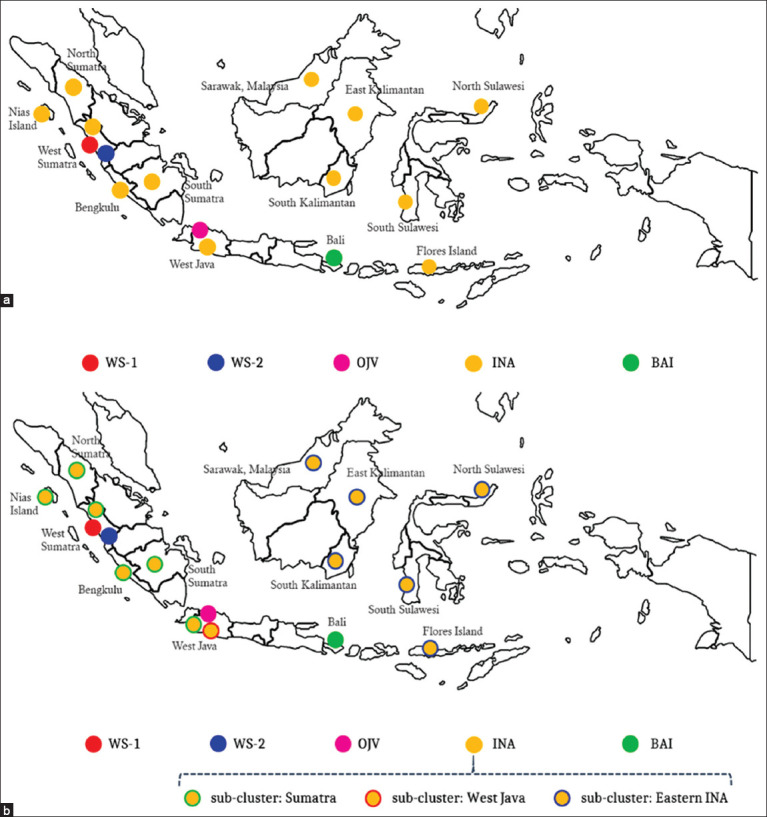
Geographical distribution of the rabies virus cluster (a) and sub-cluster (b) in Indonesia based on the N gene sequences.

The INA cluster consisted of 46 RABV isolated in 1997–2003, 2008–2010, and 2016–2021 (Figure-2). The INA cluster is the largest Indonesian RABV and originates from the western and eastern parts of Indonesia, namely, the islands of Sumatra, Java, Sulawesi, Kalimantan, and East Nusa Tenggara (Figure-3). Geographically, the INA cluster is divided into three subclusters, namely, the Sumatra, West Java, and Eastern INA subclusters (Figures-1 and 3).

RABV from Bali (29 isolates) formed a separate BAI cluster. These viruses were isolated during the rabies outbreak on the island of Bali in 2008–2010. The OJV cluster is RABV originating from Jakarta and West Java, which was isolated in 1989. The OJV cluster is genetically different from other viruses from West Java isolated between 1997 and 2003 and between 2008 and 2009 (they belong to the West Java subcluster of INA clusters).

**Figure-4:**
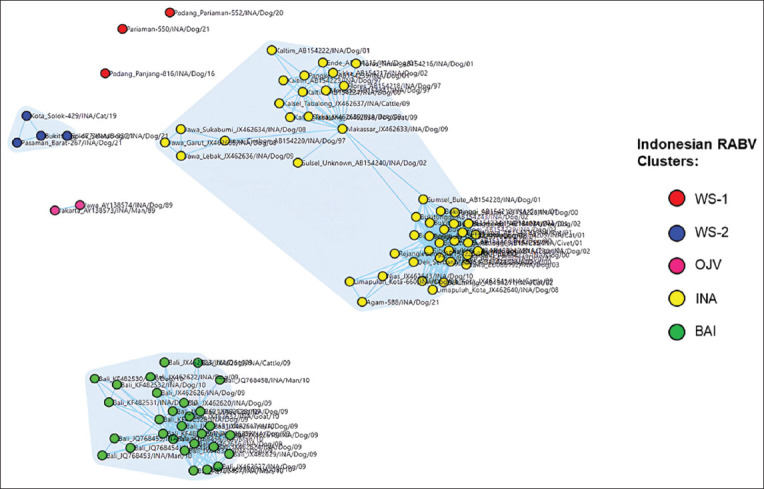
Possibility of local transmission pathways among rabies virus clusters in Indonesia.

Analysis using MicrobeTrace (Centers for Disease Control and Prevention [CDC], USA) showed the possibility of local transmission pathways occurring in the Sumatra, West Java, and eastern INA subclusters (Figure-4). The Sumatra subcluster includes all RABVs from the island of Sumatra. In contrast, the West Java subcluster includes viruses isolated from the West Java Province. The eastern INA subcluster includes RABV isolated from the islands of Kalimantan, Sulawesi, and Flores-East Nusa Tenggara Province. Local transmission pathways are thought to also occur in RABV, which was isolated from Bali as a separate cluster.

### Genetic relatedness of Indonesian RABV to other viruses in the world

**Figure-5:**
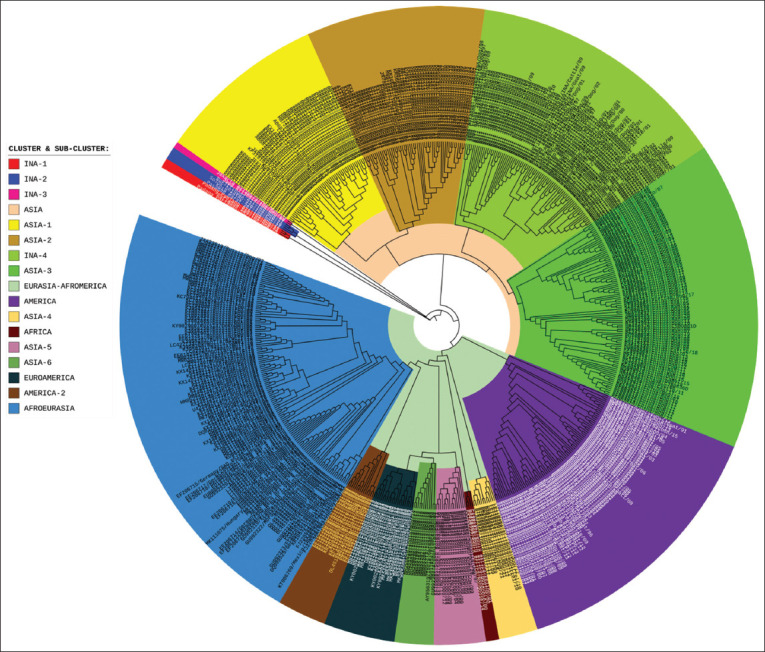
Cladogram of Indonesian rabies virus (n = 86) compared to other viruses around the world (n = 507) based on the nucleoprotein (N) gene sequences.

The phylogenetic tree for RABV in the world (Figure-5) is divided into five clusters, namely, INA-1, INA-2, INA-3, ASIA, and EURASIA-Afromerica. The INA-1 to INA-3 clusters are the same cluster with the names WS-1, WS-2, and OJV. Meanwhile, the INA cluster (Figure-1) is grouped into the INA-4 subcluster as part of the ASIA cluster.

The ASIA cluster consists of four subclusters, namely, ASIA-1, ASIA-2, ASIA-3, and INA-4. The ASIA-1 subcluster is RABV isolated from Taiwan (a province of China), China, Japan (Eastern Asia), and the Philippines (Southeast Asia). The ASIA-2 subcluster comprises RABV isolated from Southeast Asia, including Cambodia, Laos, Myanmar, and Thailand. Meanwhile, INA-4 is an Indonesian RABV that forms a subcluster. The ASIA-3 subcluster represents RABV from most regions in China and is genetically different from Chinese RABV in the ASIA-1 subcluster.

**Figure-6:**
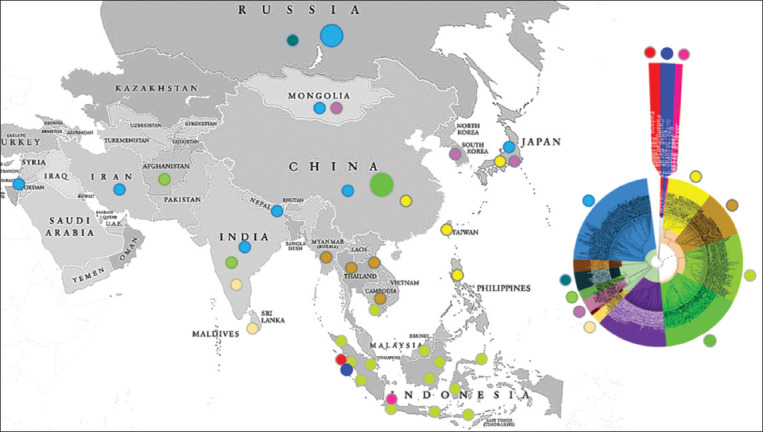
Geographical distribution of the rabies virus cluster in the Asian continent based on the N gene sequences.

The EURASIA-AFROAMERICA cluster consists of eight subclusters: ASIA-4 to ASIA-6, AMERICA, AMERICA-2, AFRICA, EUROAMERICA, and AFROEURASIA. Several RABVs from the Asian continent have closer genetic relatedness with the EURASIA-AFROAMERICA cluster, namely, the ASIA-4, ASIA-5, and ASIA-6 subclusters. The ASIA-4 subcluster comprises RABV isolated from India and Sri Lanka (Southern Asia). The ASIA-5 subcluster is RABV from South Korea, Japan, and Mongolia (Eastern Asia). The ASIA-6 subcluster is RABV from Afghanistan and India (Southern Asia). Other RABVs from Asia are genetically grouped into the AFROEURASIA subcluster (Figures-5 and 6), which are viruses isolated from western Asia (Israel), Southern Asia (Iran, India, Nepal), and Eastern Asia (Japan).

## Discussion

### Biodiversity and geographic distribution of Indonesian RABV

Indonesian RABV has shown considerable genetic diversity since three new clusters were identified, namely, WS-1, WS-2, and BAI (Figure-1). In Indonesia, RABV has a close genetic similarity and is grouped into the INA cluster. The INA cluster has been identified during three periods of RABV isolation in Indonesia, namely, 1997–2003, 2008–2010, and 2019–2021 (Figure-2). The findings from the INA cluster from 1997 to 2021 showed that the virus is genetically maintained throughout its life cycle over a long period in Indonesia. The INA cluster can be separated into three subclusters, namely, the Sumatra, West Java, and Eastern INA subclusters, which are related to their geographical distributions (Figure-3). This is consistent with the previous studies that have analyzed the genetic diversity of RABV from the islands of Sumatra, Java, Kalimantan, Sulawesi, and Flores-East Nusa Tenggara [9].

RABV, isolated from the Bali islands, is a new cluster that was first discovered in 2008. The virus was isolated from a rabies outbreak on the Bali islands and is genetically distinct from other clusters in Indonesia. The evidence in this study is similar to previously reported phylogenetic tree analyses [11, 12]. Geographically, RABV from Bali spread to a limited extent only on the island. Meanwhile, only viruses belonging to the INA cluster were found during the same period in several adjacent islands in Indonesia (Figure-3). This finding refutes previous claims that RABV from Bali is in the same cluster as viruses from Sulawesi, Flores-East Nusa Tenggara, or Kalimantan, as reported by Dibia *et al*. [12].

In 2016–2021, there was another surprise with the discovery of new clusters of Indonesian RABV originating from West Sumatra Province, namely, WS-1 and WS-2. These new clusters are genetically different from the Indonesian RABV isolated in the previous period. Thus, in the West Sumatra Province, three distinct RABV clusters were observed (Figures-2 and 3). The exact origin of the new clusters is not yet known. However, this evidence shows a progressive dynamic of the emergence of new virus variants that form different clusters. Therefore, comprehensive monitoring, control, and eradication must be performed to prevent a wider spread.

One interesting aspect that needs to be reviewed in virus control is the disappearance of RABV from the OJV cluster that originated in 1989 and was isolated from Jakarta and West Java. Jakarta has been declared a rabies-free area, whereas the surrounding area (West Java province) is still a rabies-endemic area. However, RABV, which is genetically closely related to the OJV cluster, was never found again between 1990 and 2021. Thus, this cluster is considered extinct. This is supported by evidence that the RABV identified in the West Java Province currently belongs to the INA cluster, isolated in the 1997 and 2008–2009 periods.

This evidence invalidates the assumption of Susetya *et al*. [9] that RABV from the West Java Province, which was isolated in 1997, was genetically the same as the virus isolated in 1989 (OJV). RABV originating from Jakarta and West Java Province in 1989 was genetically different from the RABV from West Java Province isolated in 1997 and 2008–2009. This finding is interesting because the first RABV identified throughout Indonesia came from the West Java Province in the 1890s [22] and is still present. Unfortunately, there is no known genetic information for Indonesian RABV before 1989.

### Probable transmission pathways of Indonesian RABV

Estimating the transmission pathway of RABV in Indonesia must consider several factors given the geographical condition of Indonesia, which consists of thousands of islands. The first is the close genetic relatedness between the virus in the outbreak area and other areas. The second is the history of animal traffic delivery or the traditional route for animal crossings that has been known for a long time. The third is the history of the appearance of the first rabies case in a certain area.

The new RABV cluster discovered on the Bali islands is thought to have originated from Kalimantan and Sulawesi [10–12], which is considered inappropriate for several reasons. First, the genetic relationship between RABV and Bali differs considerably from viruses from the islands of Kalimantan and Sulawesi. Second, geographically (Figure-3), the distribution of viruses from the BAI and INA clusters is also different. Each exhibits high-risk contact networks that occur locally in each cluster (Figure-4). This phylogeographic analysis indicates that the Bali RABV uniquely circulates only within the Bali Islands. Thus, the origin of this virus is still unknown and might be from the Bali Islands. Thus, this refutes the speculations of several scientists who reported that RABV in Bali originated from the islands of Kalimantan or Sulawesi.

Claims that RABV infects dogs in the Bali Islands, probably brought by fishermen from Sulawesi Island as happened on Flores-East Nusa Tenggara [10], must be questioned. First, a distant genetic relationship exists between RABV from the islands of Bali and Sulawesi. Conversely, genetically, RABV from the islands of Sulawesi and Flores-East Nusa Tenggara is closely related and remains in the same subcluster. Second, historically, the traditional pathway for animals crossing between the islands of Sulawesi, East Nusa Tenggara, and Kalimantan has long been known in Indonesia but not for the Bali islands. Third, based on the history of the rabies cases discovered in Indonesia, RABV was first reported in West Java and the East Coast of Sumatra in the 1890s. At the end of that period, it was reported to have spread to the Sulawesi Islands [22]. Subsequently, between 1900 and 1916, rabies cases were reported on the Kalimantan Islands and other parts of Java, Sumatra, and Sulawesi [22]. This confirms that RABV from the provinces of West Java, Sumatra, Sulawesi, and Kalimantan has been maintained for hundreds of years and remains today.

Genetically, RABV isolated from the four regions is closely related. Therefore, it is estimated that transmission between the four regions has occurred in the past, and the virus has remained. The rabies outbreak on the island of Flores-East Nusa Tenggara began in 1997 [22]. It was suspected that the transmission from the island of Kalimantan or Sulawesi to Flores seemed more accepted, as supported by evidence of RABV’s genetic similarity. Conversely, the speculation of the transmission from the island of Kalimantan or Sulawesi to the Bali Islands does not yet have scientific evidence. This should be accepted because the viruses were genetically different. In the same year, RABV was found in the INA cluster on Sumatra, Java, Kalimantan, Sulawesi, and East Nusa Tenggara islands. This indicates that RABV in Bali differs from the virus found in other parts of Indonesia when the outbreak occurred in Bali.

The close genetic relationship between RABV from the West Java Province and viruses from the island of Sumatra is due to the delivery of dogs between the two islands that have occurred since the 1890s, especially hunting dogs [23]. These dogs originated in the West Java Provinces in the areas of Garut, Sumedang, and Sukabumi [23]. This explains why the genetics of RABV in West Java Province are similar to those of viruses from Sumatra, Kalimantan, Sulawesi, and Flores-East Nusa Tenggara. The mobility of ongoing dog delivery is at risk of causing the entry of the West Java subcluster into the island of Sumatra. However, the discovery of three new clusters from the West Sumatra Province became interesting to further trace due to the potential for its spread to other regions. This is because the viruses (isolated in the 2016–2021 period) have never been reported in other regions in Indonesia.

### Genetic diversity of Asian RABV

Asian RABV that is genetically close to the virus from Indonesia is from China (ASIA-3 cluster). However, RABV from China also has another variant, the ASIA-1 cluster, along with viruses from Taiwan, Japan, and the Philippines. The RABV of Cambodia, Laos, Myanmar, and Thailand formed a separate cluster, ASIA-2, which was genetically different from the virus from Indonesia. However, in this study, it is known that some RABV from Cambodia has a close genetic relationship to the virus from Indonesia. The findings for the ASIA-2 cluster are the same as those from a study conducted in China [24].

RABV from the Southeast Asia region, which is genetically close to a virus from Indonesia, is an isolate from Malaysia. Rabies virus from Sarawak (Malaysia) is genetically very close to the virus from East Kalimantan (Indonesia). This evidence is also supported by previous research in Malaysia [8], which showed that the virus from Sarawak is in a group with the virus from Indonesia. Still, the virus from Peninsular Malaysia is in a group of viruses from Thailand, Vietnam, and others [8]. Thus, Indonesian RABV generally tends to be similar to the virus from Sarawak and China (ASIA-3 cluster).

Therefore, the ASIA-1, ASIA-2, ASIA-3, and Indonesia (Southeastern and China) clusters are typical of Asian RABV. This finding is similar to a previous report on RABV study from China [24]. RABV of Southern Asia (Afghanistan, India, Iran, Nepal, and Sri Lanka), Western Asia (Israel), and several Eastern Asia (South Korea, Japan, and Mongolia) clusters are considered closer to the virus from the EURASIA-AFROAMERICA cluster. The EURASIA-AFROAMERICA cluster in this study is similar to the Cosmopolitan lineage, with ancestral roots in Europe in the 18^th^ century before its widespread dispersal to Asia, Africa, and the Americas due to European exploration and colonization [25].

Interestingly, RABV from Indonesia and Asia has different clusters with virus strains for vaccines, such as CVS, Pasteur, SAD1, SAG2, and Pitman Moore. This indicates that the field viruses from Indonesia, China, and Southeastern Asia are genetically different from the standard virus commonly used for vaccine manufacturing based on the sequence of the N gene. However, this is not an issue. The N gene, which plays a role in encapsulation and protection of RNA from endogenous ribonuclease activities, regulation of transcription, and replication, is involved in the induction of protective immunity against rabies and rabies-related viruses and has an important role in the T helper response, particularly against challenges from lyssavirus, which is antigenically distant from the vaccine strains [26–29].

## Conclusion

The RABV in Indonesia is divided into five clusters. The isolates from the West Sumatra Province in 2016–2021 were found to be a new cluster genetically distant from other Indonesian viruses.

## Authors’ Contributions

NC, YF, and IR: Conceptualized the study, data acquisition and analysis, and reviewed the manuscript. SS and EP: Conceptualized the study and reviewed the manuscript. DTS: Data acquisition, analysis, and drafted and revised the manuscript. All authors have read, reviewed, and approved the final manuscript.
